# Psychological impact of an epidemic/pandemic on the mental health of healthcare professionals: a rapid review

**DOI:** 10.1186/s12889-020-09322-z

**Published:** 2020-08-12

**Authors:** Suzannah Stuijfzand, Camille Deforges, Vania Sandoz, Consuela-Thais Sajin, Cecile Jaques, Jolanda Elmers, Antje Horsch

**Affiliations:** 1 Institute of Higher Education and Research in Healthcare (IUFRS), University of Lausanne, Route de la Corniche 10, 1010 Lausanne, Switzerland; 2 Medical Library, Lausanne University Hospital and University of Lausanne, Rue du Bugnon 46, 1011 Lausanne, Switzerland; 3 Department Woman-Mother-Child, Lausanne University Hospital, Avenue Pierre-Decker 2, 1011 Lausanne, Switzerland

**Keywords:** Epidemic, Pandemic, COVID-19, Mental health, Healthcare professionals, Interventions, Review, Prevention, Intervention, Outbreak

## Abstract

**Background:**

Epidemics or pandemics, such as the current Coronavirus Disease 2019 (COVID-19) crisis, pose unique challenges to healthcare professionals (HCPs). Caring for patients during an epidemic/pandemic may impact negatively on the mental health of HCPs. There is a lack of evidence-based advice on what would be effective in mitigating this impact. Objectives: This rapid review synthesizes the evidence on the psychological impact of pandemics/epidemics on the mental health of HCPs, what factors predict this impact, and the evidence of prevention/intervention strategies to reduce this impact.

**Method:**

According to rapid review guidelines, systematic searches were carried out in Embase.com, PubMed, APA PsycINFO-Ovid SP, and Web of Science (core collection). Searches were restricted to the years 2003 or later to ensure inclusion of the most recent epidemic/pandemics, such as Severe Acute Respiratory Syndrome (SARS). Papers written in French or English, published in peer-reviewed journals, and of quantitative design using validated measures of mental health outcomes were included. Of 1308 papers found, 50 were included. The full protocol for this rapid review was registered with Prospero (*reg.no.* CRD42020175985).

**Results:**

Results show that exposed HCPs working with patients during an epidemic/pandemic are at heightened risk of mental health problems in the short and longer term, particularly: psychological distress, insomnia, alcohol/drug misuse, and symptoms of posttraumatic stress disorder (PTSD), depression, anxiety, burnout, anger, and higher perceived stress. These mental health problems are predicted by organizational, social, personal, and psychological factors and may interfere with the quality of patient care. Few evidence-based early interventions exist so far.

**Discussion:**

HCPs need to be provided with psychosocial support to protect their mental wellbeing if they are to continue to provide high quality patient care. Several recommendations relevant during and after an epidemic/pandemic, such as COVID-19, and in preparation for a future outbreak, are proposed.

## Background

Epidemics or pandemics, such as the current COVID-19 crisis, pose a significant threat to public health. This sudden outbreak of a novel, highly contagious disease, is unpredictable and associated with high morbidity and mortality rates [1]. An epidemic (or outbreak) is the “occurrence in a community or region of cases of an illness … clearly in excess of normal expectancy” [2] , p. 3, and a pandemic (or large scale outbreak) is “a large epidemic”, “best reserved for infectious diseases.” [3] , p.1020. Compared to other large-scale disasters, epidemics/pandemics pose unique challenges to HCPs, as the treatment course is often yet unknown, social isolation is required following presentation of first symptoms, and frontline HCPs not only fear for the safety of their patients, but also for their own health, and that of their close family members. Furthermore, many HCPs are suddenly required to carry out unfamiliar tasks in an unfamiliar area of care, such as high-risk, high-intensity units, all of which are likely to be associated with elevated levels of psychological distress [4]. These characteristics of an outbreak reduce the availability of social support, including support from their colleagues and their family, which is known to buffer the negative impact of stress [4].

### Why is this review needed?

Caring for patients during an epidemic/pandemic may impact negatively on the mental health of HCPs [5, 6]. While studies on this impact exist, this literature has yet to be updated and fully synthesized alongside a review of potential risk and protective factors. Understanding this mental health impact would sensitize policy makers and governance bodies about the importance of considering the mental health needs of HCPs in the preparations for, during, and in the aftermath of such outbreaks. Furthermore, there is a lack of evidence-based advice on what would be effective in mitigating this impact, calling for a synthesis of the evidence on prevention/intervention strategies.

We therefore conducted a rapid review on the psychological impact of pandemics/epidemics on the mental health of HCPs, what factors may protect or increase the risk of this impact and what evidence there is for prevention/intervention strategies to reduce this impact.

## Methods

The full protocol for this rapid review was registered with Prospero (*reg.no.* CRD42020175985). A rapid review is defined as a form of synthesis that streamlines or omits methods for a systematic review in order to produce evidence for stakeholders [7]. Therefore, the number of reviewers conducting each phase of the screening differed from that of a traditional systematic review and no formal study quality evaluation took place (see C Garritty, G Gartlehner, C Kamel, V King, B Nussbaumer-Streit, A Stevens, C Hamel and L Affengruber [7] for guidelines). However, a rapid review was deemed the method of choice in order to support decision makers in a timely manner on how the mental health of their HCPs during the current COVID-19 crisis can be protected.

### Search strategy and selection criteria

Following rapid review guidelines C Garritty, G Gartlehner, C Kamel, V King, B Nussbaumer-Streit, A Stevens, C Hamel and L Affengruber [7], systematic searches were carried out on the 22nd March 2020 on the databases Embase.com, PubMed, APA PsycINFO - Ovid SP, Web of Science (core collection). An additional search was performed in Google Scholar, followed by citation tracking of included studies. Searches were restricted to the years 2003 or later, ensuring inclusion of the most recent epidemic/pandemics, such as SARS. The search was based on a combination of terms related to “healthcare professional” (e.g., “healthcare provider”), “disease outbreak” (e.g., “pandemic”) and “mental health” (e.g., “depression”). It included (but was not limited to) the following epidemics/pandemics that occurred from 2003 onwards: COVID-19, severe acute respiratory syndrome (SARS), Middle East respiratory syndrome (MERS), influenza pandemic (H1N1), avian influenza (H5N1), and West Nile Fever (see Supplementary Materials: Additional file 1 for the full search algorithms).

For inclusion, papers had to be written in French or English, published in peer-reviewed journals, and present quantitative data including validated measures of mental health outcomes. Measures were judged to be valid if there was psychometric information available confirming their validity and reliability. Modified versions of validated measures were accepted if the modification entailed adapted instructions for a specific scenario/trauma/population. Intervention studies were included if the design allowed the assessment of the effectiveness of the intervention on mental health outcomes. Studies were included when HCPs worked directly with infected/suspected patients in hospitals or in communities during the outbreak (exposed). Mixed methods studies were included if quantitative data could be separated from qualitative date. Studies did not have to contain a control group for inclusion. Conference abstracts, opinion pieces, editorials, and letters were excluded, as were (reviews of) qualitative studies. Titles, abstracts and then full texts were screened by two researchers. Where the researchers were unsure of eligibility, the paper was passed through to the next phase of screening to allow further scrutiny. For each accepted article after full-text screening, two researchers carried out data extraction at different times, and a third one checked for and resolved any discrepancies. All journals of accepted papers were verified as being peer-reviewed journals through Ulrich’s Global Serials Directory, or on the website of the journal by a specialist librarian.

## Results

**Fig. 1:**
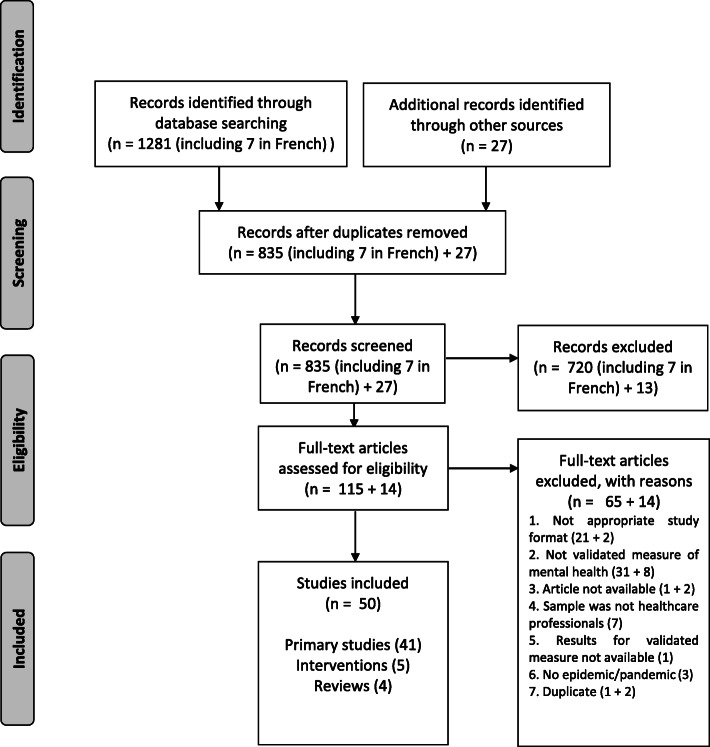
Prisma flowchart of Study Selection

Figure 1 depicts the screening and eligibility checking process and details the numbers of papers included and excluded at each phase, including reasons for exclusion for the full-text screening phase. As can be seen in Fig. 1, of 1308 papers found, 50 were included in this review. The characteristics of studies that met our inclusion criteria are presented in Table 1. Across the manuscript, as in Table 1, long-term effects are those reported in study as measured 6 months or longer after the outbreak.

**Table 1:** Study Characteristics of Accepted Studies

**Primary Studies**								
**First author (year)**		**Country (disease outbreak)**	**Timepoint (design)**	**Sample**			**Mental health outcomes**	**Measures**
AO Chan and CY Huak [8]^†^		Singapore (SARS)	Concurrent (Cross-sectional)	661 HCPs (106 SARS exposed HCPs and 555 non exposed HCPs)			PTSD Psychological Distress	IES GHQ-28
SS Chan et al. [9]^◈^		Hong Kong (SARS)	Concurrent (Cross-sectional)	1470 nurses			Psychological health	SARS NSQ
CS Chen et al. [10]^◈^		Taiwan (SARS)	Concurrent (Cross-sectional)	128 nurses (42 control, 21 conscripted and 65 high-risk nurses)			PTSD Psychological symptoms	IES SCL-90-R
NH Chen et al. [11]^†^		Taiwan (SARS)	Concurrent (Longitudinal)	172 (90 SARS exposed HCPs and 82 non HCPs)			Social support	MOS SF-36
MY Chong et al. [12]^†^		Taiwan (SARS)	Concurrent (Cross-sectional)	1257 HCPs			PTSD Psychological Morbidity	IES CHQ
SE Chua et al. [13]^◈^		Hong Kong (SARS)	Concurrent (Cross-sectional)	613 (271 HCPs from SARS units and 342 healthy control subjects)			Perceived stress	PSS-10
L Fiksenbaum et al. [14]		Canada (SARS)	Concurrent^1^ (Cross-sectional)	333 nurses			Burnout (emotional exhaustion) State anger	MBI-EE STAXI
P Goulia et al. [15] ^†^		Greece (A/H1N1)	Concurrent (Cross-sectional)	469 HCPs			Psychological distress	GHQ-28
D Ji et al. [16]		Sierra Leone (Ebola)	Concurrent (Longitudinal)	161 (59 local medical staff; 21 local logistic staff; 22 local medical students; 41 Chinese medical staff and 18 Ebola survivors)			Psychological symptoms (Global severity index, obsession-compulsion)	SCL-90-R
JS Kim and JS Choi [17]		South Korea (MERS)	Concurrent (Cross-sectional)	215 nurses from emergency department (119 MERS-exposed nurses and 96 MERS non-exposed nurses)			Burnout Job stress	OLBI Parker and DeCotiis scale
D Koh et al. [18]^†◈^		Singapore (SARS)	Concurrent (Cross-sectional)	10,511 HCPs			PTSD	IES
WJ Lancee et al. [19]^†^		Canada (SARS)	Long (Cross-sectional)	139 HWCs			Axis I diagnosis excluding the psychosis and PTSD PTSD Burnout (Emotional exhaustion)	SCID CAPS and IES MBI-EE
AM Lee et al. [20]		Hong Kong (SARS)	Concurrent Long (Longitudinal)	79 SARS patients (49 non–HCPs and 30 HCPS) 96 SARS survivors (63 non–HCPs and 33 HCPS)			Perceived Stress Perceived Stress Anxiety and Depression PTSD Psychological Distress	PSS-10 PSS-10 Subscales of DASS-21 IES-R GHQ-12
SM Lee et al. [21]		South Korea (MERS)	Concurrent (Longitudinal)	358 hospital staff (185 doing MERS-related tasks and 173 not doing MERS-related tasks)			PTSD	IES-R
M Lehmann et al. [22]		Germany (Ebola)	Concurrent (Cross-sectional)	86 (42 internal medicine staff; 32 Ebola patient treatment staff and 12 research laboratory staff)			Health-related quality of life Generalized anxiety disorder; Depression Fatigue	SF-12 GAD-7 Depression module of the PHQ-9 Fatigue subscale of the FACIT
L Li et al. [23]		Liberia (Ebola)	Concurrent^2^ (Cross-sectional)	52 HCPs			Psychological health (Obsessive compulsive symptoms)	SCL-90-R (obsessive-compulsive dimension)
CY Lin et al. [24]^†^		Taiwan (SARS)	Concurrent^3^ (Cross-sectional)	92 HCPs (66 emergency department staff and 26 psychiatric ward staff)			PTSD Psychiatric morbidity	DTS-C CHQ-12
X Liu et al. [25]^†^		China (SARS)	Long (Cross-sectional)	549 hospital workers			Depressive symptoms PTS symptoms	CES-D IES-R
YC Lu et al. [26]^†^		Taiwan (SARS)	Concurrent (Cross-sectional)	127 HCPs (24 physicians, 49 nurses and 54 other HCPs)			Psychiatric morbidity	CHQ
FW Lung et al. [27]^†^		Taiwan (SARS)	Concurrent Long (Longitudinal)	127 HCPs (24 physicians, 49 nurses and 54 otherHCPs) *(this is a follow-up of Lu* et al.*, 2006)*			Psychiatric morbidity	CHQ
IWC Mak et al. [28]		Hong Kong (SARS)	Long (Cross-sectional)	90 SARS survivors among which 27 HCPs and 63 non-HCPs			PTSD	IES-R
Z Marjanovic et al. [29]^◈^		Canada (SARS)	Concurrent (Cross-sectional)	333 nurses			Burnout (Emotional exhaustion) state anger	MBI-EE STAXI
K Matsuishi et al. [30]^†^		Japan (H1N1)	Concurrent^4^ (Cross-sectional)	1625 hospital staff (218 medical doctors, 864 nurses, and 543 others)			PTSD	IES
R Maunder [31] ^◈†^		Canada (SARS)	Concurrent (Cross-sectional)	1557 HCPs (430 nurses)			PTSD	IES
RG Maunder et al. [32]^†◈^		Canada (SARS)	Long (Longitudinal)	Survey A: 769 HCPs (587 SARS exposed HCPs and 182 SARS non exposed HCPs) Survey B: 187 HCPs			PTSD Burnout (emotional Exhaustion) Maladaptative coping;	IES MBI-EE WCQ – (escape-avoidance, self-blame, confrontative coping subscales)
GM McAlonan et al. [33]^†◈^		Hong Kong (SARS)	Concurrent Long (Longitudinal)	176 HCPs (106 high risk HCPs and 70 low risk HCPs) 184 HCPs (71 high risk HCPs and 113 low risk HCPs)			Perceived stress Anxiety, depression and stress PTS symptoms	PSS-10 DASS-21 IES-R
LA Nickell et al. [34]^†◈^		Canada (SARS)	Concurrent (Cross-sectional)	510 HCPs			emotional distress	GHQ-12
JS Park et al. [35]		South Korea (MERS)	Concurrent (Cross-sectional)	187 nurses			Mental health Perceived stress	SF-36 form (mental health subscale) PSS-10
DH Phua et al. [36]^†^		Singapore (SARS)	Long (Cross-sectional)	96 HCPs (38 doctors and 58 nurses) *(from the method looks like the same sample as Tham* et al. *(2004). However, this is not stated in the study.)*			psychiatric morbidity PTSD (psychological reactions) Coping strategies	GHQ-28 IES COPE
E Poon et al. [37]^†◈^		Hong Kong (SARS)	Concurrent (Cross-sectional)	1926 hospital staff (534 high risk hospital staff and 1392 low risk hospital staff)			Burnout (emotional exhaustion) Anxiety	MBI-EE C-STAI
K Sim et al. [38]^◈^		Singapore (SARS)	Concurrent^5^ (Cross-sectional)	277 HCPs (97 high risk HCPs and 180 low risk HCPs)			PTS symptoms Psychiatric morbidity Coping	IES-R GHQ-28 Brief COPE questionnaire
H Son et al. [39]		South Korea (MERS)	Concurrent (Cross-sectional)	280 hospital staff (153 HCPs and 127 non-HCPs)			Coping ability PTSD	K-CD-RISC IES-RK
R Styra et al. [40]^†◈^		Canada (SARS)	Concurrent (Cross-sectional)	248 HCPs (160 high risk HCPs and 88 low risk HCPs)			PTS symptoms	IES-R
T-P Su et al. [41]		Taiwan/ SARS	Concurrent (Longitudinal)	102 nurses (70 nurses from SARS units and 32 nurses from non-SARS units)			Anxiety Depression PTS symptoms Sleep disturbance (insomnia)	STAI BDI DTS-C DSM IV and PSQI
H Sun and X Ren [42]		China (SARS)	Concurrent (Cross-sectional)	73 HCPs (35 infected HCPs and 38 uninfected HCPs)			Mental health	SCL-90 Chinese version
CW Tam et al. [43]^†◈^		Hong Kong (SARS)	Concurrent (Cross-sectional)	Study design			Psychological morbidty	GHQ-12 Chinese version
KY Tham et al. [44]		Singapore (SARS)	Long (Cross-sectional)	Cross-sectional^2b^			Psychiatry morbidity PTS symptoms	GHQ-28 IES
S Verma et al. [45]^†◈^		Singapore (SARS)	Concurrent^6^ (Cross-sectional)	Cross-sectional^2b^			Psychological distress PTS symptoms	GHQ-28 IES
TW Wong et al. [46]^†◈^		Hong Kong (SARS)	Concurrent^7^ (Cross-sectional)	Cross-sectional^2b^			Coping strategies	Brief COPE questionnaire
P Wu et al. [47]^†◈^		China (SARS)	Long	Longitudinal^1b^			PTS symptoms	IES-R
H Xiao et al. [48]		China (COVID-19)	Concurrent (Cross-sectional)	Cross-sectional^2b^			Anxiety Sleep (quality) Stress	SAS PSQI SASR
**Intervention Studies**								
**Author (year)**	**Sample size**	**Country**	**Cross-sectional**^**2b**^	**Brief description of intervention**	**Impact on Mental Health (yes/no)**	**Which MH outcome?**	**Format of intervention**	**Timing of intervention**
R Chen et al. [49]^◈^	116	Taiwan	Cross-sectional^2b^	SARS prevention programme (based on information provided by WHO and CDC): In-service training, manpower allocation, gathering sufficient protective equipment, and establishment of a mental health team for patients and professionals	yes	Anxiety Depression Sleep quality	No information	Before first patient with SARS was seen
R Marrs et al. [50]	31	USA	Longitudinal^1b^	High consequence infectious diseases training using interprofessional simulation and TeamSTEPPS (based on Jeffries Simulation Theory): simulation of real life events such as patients vomiting, bleeding, having diarrhea, or respirator battery dying when caring for patients with a highly infectious disease	yes	State anxiety	2 computerised simulation sessions including interprofessional TeamSTEPPS training	Before disease outbreak
RG Maunder et al. [51]	158	Canada	Cross-sectional^2b^	Computer-assisted resilience training (interactive reflective exercises)	yes	Coping strategies: problem-solving and seeking support	Computer-assisted interactive reflective exercises of varying length: 1.75 h, 3 h and 4.5 h	Before disease outbreak
M Sijbrandij et al. [52]	408	Sierra Leone	Cross-sectional^2b^	One-day PFA training: (1) explaining important terms (mental health, mental disorder, psychosocial support and psychosocial disorder); (2) understanding reactions to traumatic and stressful events; (3) understanding PFA; (4) understanding sources and signs of stress; (5) self-care; (6) providing PFA-prepare for your role, look, listen and link; (7) ending your assistance; (8) practicing PFA with role-play	no	Professional quality of life: burnout and compassion fatigue	One-day training	Acute aftermath of disease outbreak
S Waterman et al. [53]	3273	Sierra Leone	Cross-sectional^2a^	CBT–based group intervention for HCPs with MH symptoms. Phase 1: PFA (discussion of challenges linked with work and the impact of this, ways of coping, and their achievements). Phase 2: Psychoeducation: information about a specific mental health problem and discussion of coping strategies based on behavioural and cognitive approaches (self-help). Phase 3: group CBT: behavioural activation, decreasing avoidance, problem solving, and coping with anxiety.	yes	PTSD, depression, anxiety, sleep, perceived stress, anger, relationship problems	Stepped intervention: 2-h workshop on psychological first aid + 2-h workshop on psychoeducation + 6 weekly sessions of brief CBT group programme	Towards the end of disease outbreak
**Reviews**								
**First author (year)**		**Disease outbreak**	**Sample**	**Design**	**Mental health outcomes**			
SK Brooks et al. [6]^◈^		SARS	HCPs	Cross-sectional	Psychological wellbeing; perceived stress; work/job-related stress; overall and emotional distress; panic; anxiety; PTSD; fatigue; sleep; health worries; fear of social contact; health fear; social isolation; depression; acute stress disorder; alcohol intake; anger; concerns for personal or family health; psychological support; social support; neurosis; stigmatisation; adjustment disorder; resilience; coping (including avoidance behaviour); burnout (including emotional exhaustion).			
PJ Gardner and P Moallef [54]		SARS	SARS survivors, including HCPs	Cross-sectional	Psychotic symptomatology; fear of survival; fear of infecting others; perceived stigmatisation; quality of life; psychological/emotional distress; PTSD			
M Kunin et al. [1]		SARS; H1N1	GPs	Cross-sectional	Psychological distress; anxiety; PTSD			
KJ Vyas et al. [5]^†^		SARS; H1N1	HCPs	Cross-sectionnal	Psychological distress; insomnia; alcohol/drug misuse; PTSD; depression; anxiety.			

*Note*. ^†◈^ All studies followed by these symbols were included in the review with the same symbol. Concurrent = during the outbreak; Long = reported in study as 6 months or longer after the outbreak; A-H1N1/H1N1 = influenza pandemic; *BSI* Beck Depression Inventory; *CD-RISC* The Connor-Davidson Resilience Scale; *CAPS* The Clinician-Administered PTSD Scale; *CBT* Cognitive behavioural therapy; *CDC* Centers for Disease Control; *CES-D* The Center for Epidemiologic Studies Depression Scale; *CHQ* Chinese Health Questionnaire; *CIES-R* Chinese version of Impact of Events Scale – Revised; *COPE* Coping Orientation to Problems Experienced; *COVID-19* Coronavirus disease; *C-STAI* Chinese version of the State-Trait Anxiety Inventory; *DASS-21* 21-item Depression Anxiety Stress Scales; *DSM-IV* Diagnostic and. Statistical Manual of Mental Disorders, version IV; *DTS-C* Davidson Trauma Scale Chinese version; *FACIT* Functional Assessment of Chronic Illness Therapy; *GAD-7* Generalised Anxiety Disorder Scale; *GHQ-12* General Health Questionnaire-12; *GHQ-28* General Health Questionnaire-28; *GPs* General practitioners; *HIV* Human immunodeficiency viruses; *IES* Impact of Events Scale; *IES-R* Impact of Events Scale-Revised; *IES-RK* Impact of Event Scale-Revised-Korean version; *K-CD-RICS* Korean version of the Connor-Davidson Resilience Scale; *MBI-EE* Maslach Burnout Inventory – Emotional Exhaustion; *MERS* Middle East Respiratory Syndrome; *MOS SF-36* Medical Outcome Study Short-Form 36 Survey; *HCPs* Healthcare professionals; *OLBI* Oldenburg Burnout Inventory; *PHQ-9* Patient Health Questionnaire-9; *PFA* Psychological First Aid; *PSS-10* 10-Item Perceived Stress Scale; *PSQI* Pittsburgh Sleep Quality Index; *PTS* Posttraumatic stress; *PTSD* Posttraumatic stress disorder; *RCT* Randomized Controlled Trial; *TCMPs* Traditional Chinese Medical Practitioners; *SAS* Self-Rating Anxiety Scale; *SASR* Severe Acute Respiratory Syndrome; *SARS NSQ* SARS Nurses’ Survey Questionnaire; *SCL-90* Symptom checklist; *SCL-90-R* Symptom Checklist-90-Revised; *SCID* Structured Clinical Interview; *SF-12* 12-Item Short Form Health Survey; *SF-36* 36-Item Short Form Health Survey; *STAI* State-Trait Anxiety Inventory; *STAXI* State-Trait Anger Expression Inventory; *WCQ* Ways of Coping Questionnaire; *WHO* World Health Organization

^1^According to authors [Chua et al., 2004], “data were collected between March and May 2004” (p.97) occurring one year after the SARS outbreak. However, retrospective information was collected.

^2^According to authors [Li et al., 2015], participants “were enrolled from March 1 to 10, 2015” (p.2). Please note that the Liberia outbreak was declared over by May 2015 (source = https://www.cdc.gov/about/ebola/timeline.html).

^3^According to authors [Lin et al., 2007], « Taiwan was “a SARS-affected area” from 30 April to 5 July 2003″ (p.12). Data collection “began on 5 August and ended on 11 August 2003” (p.13).

^4^According to authors [Matsuishi et al., 2012], data was collected “approximately 1 month after the peak of the H1N1 outbreak in Kobe City” (p.355).

^5^According to authors [Sim et al., 2004], “although Singapore was removed from the list of areas with recent local transmission by the World Health Organization on May 31, 2003, the operation of the fever tents at the polyclinics was not terminated until August 1, 2003. […] (T)he study instrument was distributed to the medical staff at the beginning of the week in mid-July 2003” (p.1121).

^6^According to authors [Verma et al., 2004], data were collected “about 2 months after the first case of SARS was reported in Singapore” (p.744).

^7^According to authors [Wong et al., 2005], « data were collected from late June to early July 2003 after Hong Kong was removed by the World Health Organization from the list of areas with local transmission of SARS on 23 June 2003) (p.14).

From the included papers, two systematic reviews were identified that directly contributed to the research questions. One reviewed the evidence of the impact of past outbreaks on the mental health of HCPs [5] and one reviewed the evidence for organizational and social predictors of the impact of past outbreaks on the mental health of HCPS [6]. Therefore, a summary of these systematic reviews are a focal part of this rapid review. Of the 50 accepted papers for this rapid review, 21 were included in the review of Vyas et al. [5] and 16 were included in the review of Brooks et al. [6], ten appeared in both (see Table 1). Beyond the systematic reviews, data extracted from primary studies are included in this rapid review if they are more recent than the search dates of the systematic reviews, report on mental health outcomes not covered by the first systematic review, or investigated predictors of mental health outcomes not included in the second systematic review.

### The psychological impact of an epidemic/pandemic on the mental health of healthcare professionals

A systematic review and meta-analysis [5] (including studies from 2000 to 2014) showed an impact of an epidemic/pandemic on the mental health of HCPs. This review included studies using both diagnostic tools and self-report measures with clinical cut-offs to assess mental health outcomes. Therefore, percentage prevalence’s are best interpreted as ‘probable’ percentage of cases. Effect sizes (standardised mean difference) reflect the difference between an exposed HCPs group and a control group. Thus, where a positive effect is reported, the exposed group showed higher symptom scores than the control group. In this review, *psychological distress* was assessed in 13 studies, with an average rate among exposed HCPs of approximately 40% (range: 11–75%). *Insomnia* was assessed in four studies, with an average rate among exposed HCPs of approximately 39% (range: 30–52%). *Alcohol and drug misuse* were assessed in five studies, with an average rate of approximately 13% (range: 6–21%). *Posttraumatic stress disorder* (PTSD) symptoms were assessed in 19 studies, with an average rate of approximately 21% (range: 10–33%), of whom 40% reported persistently high PTSD symptoms 3 years after exposure. Meta-analytic results showed effects were small, (SMD = 0.12, 95% CI = − 0.23 to 0.47) but not significant. *Depression* symptoms were measured in eight studies, with an average rate of approximately 46% (range: 23–74%), of whom up to 9% reported severe levels. 11% were clinically diagnosed 1 month after the disease outbreak. Meta-analytic results showed effects were moderate (SMD = 0.40, 95% CI = 0.24–0.51) and significant. *Anxiety* symptoms were assessed in fourteen studies. The average rate was approximately 45% (range: 19–77%). Meta-analytic results showed effects were small, (SMD = 0.08, 95% CI = − 0.09 to 0.25) and not significant.

Further mental health outcomes were reviewed that had not been included in Vyas et al. [5] or more recent papers (2015–2020) containing more data on the same outcomes. Table 2 contains all data related to the mentioned relationships. *Burnout* symptoms were assessed by five studies [14, 17, 29, 32, 37]. It should be noted that the sample of Z Marjanovic, ER Greenglass and S Coffey [29] is the same sample as L Fiksenbaum, Z Marjanovic, ER Greenglass and S Coffey [14]. Burnout symptoms during the outbreak were shown to be correlated with exposure [14], were significantly higher in HCPs exposed to the outbreak than in non-exposed HCPs [17, 37], and were predicted by exposure (vs non-exposure) [29]. The difference between exposed and non-exposed groups were significant over a year after the outbreak [32] and also impacted on HCPs’ ability to work. Indeed, exposed HCPs were more likely than non-exposed HCPS to work reduced hours and have more sickness absence [32], but also to show avoidant behaviour toward patients [29]. Across these five studies, there is thus accumulating evidence of the impact of an epidemic/pandemic on burnout symptoms during the outbreak, with some evidence of a long-term effects, and detrimental patient care-related behaviours during and after the outbreak.

**Table 2:** Table of results of accepted studies referred to in the manuscript, which provide evidence for the impact of pandemics/epidemics on the mental health of healthcare professionals beyond the systematic review of KJ Vyas, EM Delaney, JA Webb-Murphy and SL Johnston [5]

First author (year)	Statistical approach	Results
SE Chua, et al. [13]	Difference between HCPs and healthy controls on stress levels (no inferential test)	Stress levels for HCPs (*M* = 18.6, *SD* = 4.9) were similar to healthy control subjects (*M* = 18.3, *SD* = 5.6), but 50% higher than the normative value for the PSS-10.
Fiksenbaum et al. (2006) [14]	Correlations between contact with SARS patients, and emotional exhaustion and state anger.	Exposure amongst nurses was significantly correlated with emotional exhaustion (*r* = −.21; *p* < .001) and state anger (*r* = −.18; *p* < .001).
D Ji, et al. [16]	Difference in the psychological dimensions of the SCL-90-R between 1 week after arrival of Chinese medical staff in an outbreak zone (Sierre Leone) and 1 week after withdrawal (either Man Whitney U or t-test)	Obsessive compulsion (*M* = 1.39, *SD* = .18 vs *M* = 1.23, *SD* = .36; *p* =. 1421); depression (*M* = 1.22, *SD* = .31 vs *M* = 1.18, *SD* = .29; *p* = .5480); hostility (*M* = 1.09, *SD* = .13 vs *M* = 1.09, *SD* = .18; *p* = 1.00); paranoid ideation (*M* = 1.11, *SD* = .19 vs *M* = 1.11, *SD* = .24; *p* = 1.00) and psychoticism (*M* = 1.14, *SD* = .24 vs *M* = 1.08, *SD* = .14; *p* = 1.706).
JS Kim and JS Choi [17]	Group differences between MERS exposed vs not exposed nurses on MERS-related burnout (t-test)	Nurses exposed to infected/−suspected patients had higher MERS-related burnout scores (*M* = 3.09, *SD* = 0.48) than non-exposed nurses (*M* = 2.93, *SD* = 0.42, *p* = .013).
WJ Lancee et al. [19]	Group differences between HCPs with vs. without history of mental illness on mental disorder development (Fischer test).	A year after the outbreak, HCPs with a history of mental illness before the outbreak had higher risk of developing a new mental DSM-IV axis 1 mental disorder (18%), compared to healthcare workers without (2%, *p* = .03).
M Lehmann et al. [22]	Group differences between internal medicine staff, Ebola patient treatment staff and research laboratory staff on anxiety levels (*Test unspecified*).	Internal medicine staff, Ebola patient treatment staff and research laboratory staff did not significantly differ levels of anxiety.
IWC Mak et al., 2009. [28]	Group differences between infected HCPs and infected non HCPs on PTSD prevalence (*Test unspecified*).	Thirty months after SARS outbreak, PTSD prevalence was higher among infected HCPs (40.7%) than among infected non HCPs (19%, *p* = .031).
Z Marjanovic et al. [29]	Correlation between contact with SARS patients, and emotional exhaustion and state anger in nurses. Multiple regressions for emotional exhaustion and state anger. Correlation between avoidance behavior, and emotional exhaustion and state anger.	Contact with SARS patient was significantly correlated with emotional exhaustion (*r* = −.21; *p* < .001) and state anger (*r* = −.18; *p* < .001). Contact with SARS patients significantly predicted emotional exhaustion (*β =* −.15, *p* = .003) but did not predict state anger (*β = −*.09, *p* = .068). Avoidance behavior was significantly correlated with emotional exhaustion (*r* = .26; *p* < .001) and state anger (*r* = .33; *p* < .001).
RG Maunder, et al. [32]	Group differences between SARS exposed vs not exposed HCPs on burnout prevalence (χ^2^). Group differences between SARS exposed vs not exposed HCPs on burnout (t-test or Mann-Whitney U Test) Group differences between SARS exposed vs not exposed HCPs on face-to-face patient contact (χ^2^). Group differences between SARS exposed vs not exposed HCPs on work hours (χ^2^).	Burnout prevalence is higher in exposed HCPs (30.4%) than HCPS not exposed (19.2, *p* = .003) Exposed HCPs had significantly higher burnout scores (*Md* = 19, *IQR* = 10–29) than non- exposed HCPs (*Md* = 16, *IQR* = 9–23) Since SARS outbreak, significantly less face-to-face patient contact was reported by exposed HCPs (16.5%) compared to those who were not exposed (8.3%, *p* = .007). Since SARS outbreak, significantly less work hours was reported by exposed HCPs (8.6%) compared non exposed HCPs (2.2%, *p* = .003).
GM McAlonan et al. [33]	During outbreak: Group differences between high vs low risk HCPs on perceived stress (t-test). Comparison of symptom scores to norm (no inferential test) One year after outbreak: Group differences between high vs low risk HCPs on perceived stress (2-way ANOVA). Interaction between time and infection level tested with a 2 way ANOVA.	Perceived stress levels did not significantly differ between high vs low risk HCPs (*t*(164) = − 1.36, *p* = 0.176) although they were higher than the normative value (13). Perceived stress levels of high-risk HCPs (*M* = 18.6, *SD* = 4.9) were significantly higher than the low-risk HCPs (*M* = 14.8, *SD* = 5, *p* < .05). Change in perceived stress from 2003 to 2004 was significantly different for the 2 groups (F1,336 = 4.61, *P* < 0.05), with a general trend toward a decrease over time for low-risk HCPs and an increase for high-risk HCPs.
JS Park et al. [35]	Mediation analysis of the relationship between hardiness and mental health by perceived stress Mediation analysis of the relationship between stigma and mental health by perceived stress	The relationship between hardiness and mental health was partially mediated by perceived stress (indirect effect 0.251, Boot SE = 0.638). Where increased hardiness led to descrease stress (*B* = −.31, *SE* = .05, *p* < .001), which subsequently led to better mental health symptoms (*B* = −.81, *SE* = .13, *p* < .001). The relationship between stigma and mental health was mediated by perceived stress (indirect effect = − 0.061, Boot SE = 0.020). Where increased stigma led to increase stress (*B* = .075, *SE* = .023, *p* = .002), which subsequently led to better mental health symptoms (*B* = −.81, *SE* = .13, *p* < .001).
E Poon et al. [37]	Group differences between hospital workers who had contact with SARS patients vs no contact with SARS patients on burnout symptoms (t-test).	Hospital workers who had contact with SARS patients had significantly higher burnout symptoms (*M* = 7.3, *SD* = 5.3) than those who did not have contact with SARS patients (*M* = 5.1, *SD* = 4.7, *p* < .001).
K Sim et al. [38]	Group differences between doctors and nurses with versus without psychiatric morbidities on effort coping, in context of SARS outbreak (Mann-Whitney U Test) Group differences between doctors and nurses with versus without posttraumatic morbidities on effort coping, in context of SARS outbreak (Mann-Whitney U Test). Group differences were examined between exposed and non exposed medical staff on psychiatric symptoms (Mann-Whitney test) and posttraumatic symptoms (χ^2^), in the context of a SARS outbreak.	Doctors and nurses with psychiatric morbidities had higher scores on effort coping (*M* = 49.7, *SD* = 13.2) than doctors and nurses without psychiatric morbidity (*M* = 39.7, *SD* = 10.4, *p* < .001) Doctors and nurses with psychiatric morbidities had higher scores on effort coping (*M* = 53.4, *SD* = 13.1) than doctors and nurses without psychiatric morbidity (*M* = 40.6, *SD* = 10.9, *p* < .001). Exposed medical staff showed no difference to non-exposed staff in psychiatric symptoms (*M* = 2.6, *SD* = 4.2 vs. *M* = 2.3, *SD* = 4.4, *p* = .28) or presence of posttraumatic symptoms (7.2% vs.10.6%, *p* = .40).
TW Wong et al. [46]	Group differences between doctors, nurses and healthcare assistants on coping strategies, in context of SARS outbreak (ANOVA with post hoc analyses).	Planning was more likely to be used by doctors (*M* = 5.33, *SD* = 1.44) compared to nurses (*M* = 4.85, *SD* = 1.44, *p* < .05) and healthcare assistants (*M* = 4.42, *SD* = 1.56, *p* < .01). Behavioral disengagement was more likely to be used by nurses (*M* = 2.96, *SD* = 1.26) than doctors (*M* = 2.56, *SD* = 0.91, *p* < .01). Self-distraction was more likely to be used by healthcare assistants (*M* = 4.58, *SD* = 1.92) than doctors (*M* = 4.11, *SD* = 1.42, *p* < .05).
H Xiao et al. [48]	Assessment of the indirect pathway from social support to sleep quality via perceived stress.	The relationship between social support and sleep quality was mediated by perceived stress (*B* = −.06, *SE* = .01, *p* = .002). Where a lack of social support (*B* = .57, *SE* = .09, *p* < .001) led to an increase in perceived stress, which subsequently led to lower sleep quality (*B* = .26, *SE* = .01, *p* < .001).

Note. *HCPs* Healthcare professionals; *MERS* Middle East Respiratory Syndrome; *SARS* Severe Acute Respiratory Syndrome; *PSS-10* 10-Item Perceived Stress Scale; *PTSD* Post traumatic stress disorder

Two studies [14, 29] investigated *state anger* within the same sample. L Fiksenbaum, Z Marjanovic, ER Greenglass and S Coffey [14] showed that caring for infected patients was correlated with increased levels of state anger in HCPs during the outbreak. Z Marjanovic, ER Greenglass and S Coffey [29] found that exposure (vs non-exposure) did not predict state anger but the latter was correlated with avoidant behaviour towards patients during the outbreak. As results pertain to the same sample, evidence for an impact on state anger is weak.

Five studies [13, 20, 33, 35, 48] investigated levels of *perceived stress*. Two studies found that during the outbreak, perceived stress levels of exposed HCPs were higher than a normative value [13, 33], whereas two studies showed perceived stress was no different between exposed and non-exposed HCPs [20, 33]. However, a year following the outbreak, perceived stress was higher amongst exposed vs non-exposed HCPs and had increased over time [33]. In addition, a year following the outbreak, perceived stress was higher amongst HCPs vs non-HCPs and had increased over time for HCPs only [20]. Evidence also indicates that during a pandemic, perceived stress was a mediator between social support and sleep quality [48] and between hardiness (resilience) and stigma, respectively, and mental health [35].

Two studies [38, 46] investigated *coping strategies* during an epidemic/pandemic. One showed that, during an outbreak, HCPs with psychiatric or PTSD symptoms used maladaptive coping strategies compared with those without symptoms [38]. It should be noted that there was no difference between exposed vs non-exposed HCPs on psychiatric or PTSD symptoms [38]. Furthermore, without a pre- outbreak measure, it is unclear whether all staff were equally affected and there is thus no evidence of the effect of the outbreak. However, the size of the non-exposed sample was double that of the exposed group, raising questions of power for that test. The second study showed that during an outbreak, different groups of HCPs used different coping strategies (see Table 2) [55]. Authors stated that the sample had been exposed to the infection; however, without a comparison group or ‘pre-outbreak’ measure, it is unclear whether the use of coping strategies was affected by the outbreak. These two studies suggest that during an outbreak, HCPs may engage in maladaptive coping strategies, however, it is unclear whether use of these strategies increased due to an outbreak.

One study [28] investigating the long-term effects of an outbreak on *PTSD* symptoms found that infected HCPs had significantly higher rates of chronic PTSD (30 months post SARS) than infected non-HCPs.

One further small study found that 2% of healthcare professionals with no psychiatric history before the outbreak had a *new DSM-IV axis 1 mental disorder* within 1 year after the outbreak [19]. Further research found no differences in symptoms of *generalised anxiety disorder* assessed during the outbreak between internal medicine staff, Ebola patient treatment staff, and research laboratory staff [22]. Another study found Chinese HCPs’ symptoms of *obsession-compulsion, depression, hostility, paranoid ideation, and psychoticism* did not change from 1 week after arrival in an infected zone in Sierra Leone to 1 week after leaving. This may perhaps be explained by the fact that these HCPs were not in their own country and thus perhaps not subject to the same worries of going home and infecting families, as local staff [16]. Furthermore, when considering symptoms of obsessive compulsion, it should be noted that many of the behaviours considered symptoms may be ‘normal’ in times of an epidemic/pandemic, e.g., frequent washing of hands.

In conclusion, healthcare professionals exposed to working with patients during the COVID-19 outbreak may be at heightened risk of mental health problems, particularly, psychological distress, insomnia, alcohol/drug misuse, and symptoms of PTSD, depression, anxiety, burnout, anger, higher perceived stress, and are more likely to engage in maladaptive coping strategies.

### Predictors of psychological impact an of epidemic/pandemic on the mental health of healthcare professionals

The next section of this rapid review focuses on synthesizing the evidence on protective or risk factors with a view to informing recommendations for prevention and intervention. One systematic review synthesizing the social and occupational factors affecting the mental health of HCPs covered the literature up to 2015 and included 22 studies [6], all of which had investigated the SARS epidemic. SK Brooks, R Dunn, R Amlôt, GJ Rubin and N Greenberg [6] identified six organizational and four social factors as showing an influence on mental health outcomes. For this rapid review, no further evidence of social and organizational factors published after 2015 was identified amongst our accepted papers. Below is a brief summary of the organizational and social factors found by Brooks et al. [6] and associated data can be found in [6]. Further predictors, beyond organizational and social factors, may also influence the impact of epidemics/pandemics on mental health. Therefore, evidence for further protective and risk factors was extracted from other primary studies accepted for this rapid review. Thirteen papers were identified. Further predictors were classified as *Psychological factors* or *Personal factors*.

#### Organizational predictors [6]

*Occupational role* influenced mental health in HCPs, with those in direct contact with infected patients showing the poorest psychological outcomes. Nurses had poorer outcomes than doctors. *Specialized training and preparedness* showed as a protective factor against stress and anxiety. However, where training was perceived as inadequate, HCPs were more likely to experience symptoms of burnout and PTSD, and their symptoms often continued in the longer term. *High-risk environments* (i.e., a high risk of exposure to infected patients) were associated with higher symptoms of anxiety, stress, PTSD, alcohol consumption, burnout, and sleep problems. *Being in quarantine* was associated with higher symptoms of acute stress disorder, PTSD, and alcohol intake. The longer the quarantine, the greater an adverse effect was found on anger symptoms and avoidance behaviors.

*Job stress,* in particular where one’s ability to do one’s job was compromised, lack of control of one’s job, and being involuntary deployed to work with infected patients negatively influenced mental health outcomes. For example, those who had to involuntarily care for infected patients reported higher levels of anxiety and depression symptoms than volunteers. *Perceptions of safety threat and risk* was identified as a protective and a risk factor for mental health. Feelings of trust in equipment and infection control procedures predicted lower emotional exhaustion and state anger. Belief in the precautionary measures within the workplace decreased concerns. However, high perception of personal risk predicted PTSD symptoms.

#### Social predictors [6]

In the context of an epidemic/pandemic, *organizational support* and *family/friends support* can function as protective factors when at adequate levels. However, low levels or inadequate organizational support, inclusive of psychological support and inadequate insurance/compensation, were risk factors for mental health. *Social rejection or isolation* was associated with poorer mental health outcomes. HCPs who experienced an *impact on life* (e.g., reduced contact with family) due to the outbreak showed greater mental health problems.

#### Personal predictors

Some personal characteristics were found to increase the risk of mental health problems of HCPs during an epidemic/pandemic*.* Those who were *single* were 1.4 times more likely to have minor psychiatric disorders according to a clinical cut-off (95% *CI* = 1.02–2.0, *p* = .048) during an outbreak. However, there was no test of whether this differed between exposed and non-exposed HCPs [8]. Being single was also found to be predictive of higher depressive symptoms (*AOR* = 4.35, *95% CI* = 1.65–11.42; *p* = .0029) amongst hospital staff during an outbreak, though this test did not separate exposed from non-exposed HCPs [25]. Being single was also cited in the systematic review of [5] as being predictive of higher symptoms of psychological distress, higher depressive symptoms, and persistent PTSD symptoms. However, in one study by K Sim, PN Chong, YH Chan and WS Soon [38], being married was predictive of the presence of PTSD symptoms (*OR* = 11.43, *CI* = 1.41 to 100, *p* = .02). In another study, higher PTSD symptoms were found amongst those who lived in a *dormitory or away from their family* (*M* = 37.2, *SD* = 20.2) than those living with family (*M* = 33.6 *SD* = 19.5.5; *p* < .005) [12]. During an outbreak, more nurses who perceived stress (50.7%) additionally reported *average or poor physical health* than those who reported no stress (18.4%, *p* = .001) [9]. *Less healthcare work experience* predicted higher psychological distress symptoms in exposed HCPs (*β* = −.26, *t* = − 3.28, *p* = .001) [32]. Being a healthcare professional with a *younger age* [38] predicted the presence of PTSD symptoms during an outbreak (*OR* = .94, *CI* = 0.89 to 0.98, *p* = .007). KJ Vyas, EM Delaney, JA Webb-Murphy and SL Johnston [5] in their systematic review also identified a younger age as predictive of symptoms of anxiety, depression and PTSD, and identified *less healthcare experience* as a predictor of symptoms of psychological distress, and PTSD. KJ Vyas, EM Delaney, JA Webb-Murphy and SL Johnston [5] also reported that HCPs with a *lower household income* reported higher PTSD symptoms during an outbreak. Finally, experiencing *stigma* (social rejection, prejudice, or discrimination due to their work) as HCPs during the outbreak predicted concurrent mental health symptoms (*β* = − 0.306, *t* = − 7.2376, *p* < 0.001). This relationship was found to be mediated by perceived stress (indirect effect = − 0.061, Boot SE = 0.020) [35].

#### Psychological predictors

*Resilience (hardiness)* is a potential protective factor and was found to have both a direct and an indirect influence on mental health during an outbreak [35]. A higher resilience score directly predicted better mental health in exposed HCPs (*β* = 0.49, *t* = 4.87, *p* < 0.001). Indirectly, hardiness, was associated with decreased stress perception, and this in turn was associated with better mental health (indirect effect = 0.251, Boot SE = 0.638) [35]. *Maladaptive coping* was a risk factor, with long-term predictive effects found on symptoms of burnout (*β* = 0.29, *t* = 3.34, *p* = 0.001), PTSD (*β* = 0.31, *t* = 3.78, *p* < 0.001), and psychological distress (*β* = 0.37, *t* = 4.39, *p* < 0.001) [32]. *Fatigue* (physical and mental) predicted symptoms of poor mental (*B* = − 0.30, *SE* = 0.12, *p* = .012) and physical (*B* = − 0.53, *SE* = 0.11, *p* < .001) health during an outbreak, alongside perceived lack of knowledge of the infection [22]. Furthermore, having a *negative emotional experience* of the outbreak predicted an increased likelihood of PTSD amongst HCPs (β = .17, *p* < .01). In this study, authors state negative emotional experience influenced PTSD symptoms of non-HCPs more than HCPs, while perceived risk (of infection) affected HCPs more than non-HCPs. However, how the statistical difference in magnitude of the coefficient was carried out was unclear [39]. More HCPs showing a new onset psychiatric disorder in the long term following an outbreak had a *psychiatric disorder before the outbreak* (18%) than those without a new onset (2%; *p* = .03) [19].

Evidence for the psychological and personal factors identified in this review comes from one or two studies, suggesting preliminary rather than strong evidence. It is also not yet clear which of these factors is the most important. This preliminary evidence points towards identifying those at risk, who may benefit from prevention/intervention programs, and what preventions/intervention may wish to target to influence mental health of HCPs.

### What can be done to prevent or reduce the impact of an epidemic/pandemic on the mental health of healthcare professionals?

#### Intervention programs

Five studies [49–53] investigating the effect of preventative programs or interventions addressing mental health outcomes in HCPs were included (see Table 1 for more details about the content of the intervention and the study design). Regarding the *preventative* programs, the SARS prevention program addressed organizational, patient-care and psychological issues before HCPs saw the first infected patients and lead to an improvement in anxiety and depression symptoms, as well as sleep quality [49]. In another study, two computerised simulation sessions of real-life events linked to caring for infected patients resulted in lower state anxiety symptoms [50]. A pilot randomized controlled trial (RCT) testing varying lengths (1.75 h, 3 h and 4.5 h) of a computer-assisted resilience training (interactive reflective exercises) before the disease outbreak resulted in improved coping strategies (problem-solving and seeking support), with the medium length being optimal [51].

Regarding *early intervention* programs in the acute aftermath of the outbreak, a one-day psychological first aid training did not lead to improved professional quality of life (burnout and compassion fatigue) [52]. However, a stepped intervention introduced towards the end of the outbreak led to a decrease in symptoms of PTSD, depression, anxiety, anger, as well as perceived stress and relationship problems, and an improvement in sleep [53]. This early intervention program consisted firstly, of a two-hour workshop on psychological first aid, after which improvement in mental health symptoms was assessed. If individuals needed more, a two-hour workshop on psychoeducation was offered and again, improvement in their symptoms was evaluated. If more help was needed, then six weekly sessions of a brief cognitive behavioral therapy (CBT) group program were offered. Of note: HCPs were trained by mental health experts to carry out this stepped approach for their peers.

#### Recommendations

Please note that the following recommendations are based on the evidence of risk and protective factors, as well as intervention studies identified by this review. It is worth noting, that those based on risk and protective factors have not yet been tested for effectiveness.

##### Before the disease outbreak

An infectious disease prevention program should be put into place by individual health services but coordinated at an international level. Important elements of the program are training of HCPs, planning and allocation of staff, provision of sufficient protective equipment, and establishment of a mental health team for professionals [49]. This may also include computerized simulation training of patient care during an outbreak [50] and a computer-assisted resilience training consisting of interactive reflective exercises [51].

##### During the disease outbreak

Given the likely increase of mental health problems among HCPs, widespread screening to identify those in need of support should be carried out, as the increased stress and burden, as well as stigma experienced by HCPs may make it hard for them to actively seek help [35]. Based on the evidence of risk factors, the following groups may be in particular need of psychological support: HCPs having direct contact with infected patients [6], those that are involuntary deployed to work with infected patients [6], those with less healthcare work experience [5, 32], individuals who are single, or do not currently live with family [12, 25], of younger age [5, 32], and those with a lower household income [5]. Comparing different groups of HCPs, those who spent time in quarantine should be prioritized [6, 25].

A widespread educational campaign alerting HCPs to the possibility of experiencing mental health problems may also help to make those in need come forward for help, as well as fight the potential stigma often associated with mental health problems [35]. Assessment of a wide range of mental health outcomes and psychological distress linked to the disease outbreak [6] is recommended, particularly symptoms of insomnia, alcohol/drug misuse, PTSD, depression, anxiety, burnout, anger, and perceived stress [5, 32, 33]. For those reporting mental health problems, a three-phased stepped intervention consisting of a workshop on psychological first aid, a workshop on psychoeducation, and a brief CBT group program may be helpful [53]. In order to increase access, this intervention could be carried out by generic healthcare professionals (peers) trained by mental health specialists [53].

With regards to organizational factors, managers should increase organizational support and foster peer support [6]. HCPs should be encouraged to volunteer for working with infected patients [6], rather than be deployed. Managers should regularly provide updated information about the epidemic/pandemic and how HCPs can best protect themselves [6]. Adequate specialized training should be made available [6, 32], with personal infection control as a priority [6, 9].

##### After the disease outbreak

HCPs’ perceived risk should be screened within a few months after the disease outbreak, as this is a risk factor for mental health and occupational problems over 1 year after the outbreak [32].

## Discussion

By conducting this rapid review, we have brought together into one place: the evidence on the impact of pandemics/epidemics on the mental health of HCPs, the evidence of influencing factors on the impact pandemics/epidemics on the mental health of HCPs, and evidence on prevention/interventions to mitigate this impact. Furthermore, we have updated a previous review [5] and broadened the set of mental health outcomes. We bring an additional 10 primary studies beyond those found in the systematic reviews and an additional three papers on interventions. Previously, evidence on social and organizational risk factors had been synthesized [6] and this rapid review adds evidence on psychological and personal risk factors.

Results from this rapid review suggest that HCPs may experience an adverse impact on their mental health during an outbreak, and in the short and long term. However, there remain questions about what consequences the impact on HCPs’ mental health will have on levels broader than the individual. Firstly, it seems likely that the mental health issues evidenced here would impact patient care. However, what is not clear from the evidence available so far is whether there is something unique about an epidemic/pandemic that would compromise professional functioning, including patient care, or whether this is due to a more general impact of mental health problems in professionals (that also occurs outside the context of an epidemic/pandemic). Secondly, there may be costs at the organizational and societal levels, as HCPs suffering from the psychological impact of the epidemic/pandemic struggle to maintain their previous working hours, thus affecting staffing levels within the health system [32] and patient care [29]. What none of the reviewed studies sufficiently addresses is the issue that part of the challenge for HCPs is the increased professional demand at a time when both family stress and personal threat (to health) are also elevated.

This rapid review makes recommendations to reduce the negative impact on HCPs’ mental health from the evidence of risk and protective factors. However, there remains a lack of evidence-based interventions/preventions that can be recommended for implementation with confidence. Evaluation of these recommendations as part of their implementation would assist future preparations for disease outbreaks to reduce and prevent the impact on the mental health of HCPs.

When considering the findings and recommendations of this rapid review, several elements should be noted. The majority of the evidence from accepted primary studies is heavily reliant on cross-sectional studies assessing self-reported symptoms. No accepted study used a longitudinal design with diagnostics. While it is appreciated that this type of data is collected rapidly in a reactive fashion, researchers should consider the importance of gathering high-quality evidence of true prevalence and risk factors. There were not enough studies or details within these studies to distinguish between specific professional groups or health contexts. Consequently, we took a broad-brush approach across professions and contexts when reporting our findings. Furthermore, not all studies had a control group of a non-exposed group but only reported prevalence’s during an epidemic/pandemic. We could also consider if the risk and protective factors for HCPs identified here may apply to other key worker professions currently at risk of contact with infected members of the public e.g., teachers.

Moreover, most of the studies were conducted in Asian countries, with only two coming from Europe, eight from Canada/USA, and four from Africa. It is likely that cultural differences between these countries are associated with different nuances in the expression of psychological outcomes. Currently, studies/reviews are being published on a daily basis related to COVID-19 and by the time of publication, there will likely be a small body of papers that we were not able to include. Finally, we would like to acknowledge that solid evidence and practice guidelines about psychosocial interventions following other large-scale disasters exist, although they do not specifically target HCPs, e.g.,B Juen, R Warger, S Nindl, H Siller, MJ Lindenthal, E Huttner and S Thormar [56]. However, it is still unknown to what extent these would also be effective in response to an epidemic/pandemic and future research should investigate whether the mental health impact of (and therefore the intervention required following) an epidemic/pandemic is unique or comparable to that of other large-scale disasters.

A rapid review has some limitations [7], as discussed above. The number of databases searched, languages included, and dates searched were limited. No qualitative studies or grey literature (unpublished or non-commercial material e.g., policy statements or government reports) was included, which may have created a potential (publication) bias. Strengths of the study included strict inclusion/exclusion criteria and only accepted peer-reviewed studies that used validated measures of mental health. Further strengths of this review are that the search terms and strategies were developed in collaboration with specialist librarians and that hand searches of references from accepted full texts were conducted. Additionally, was that multiple researchers cross-checked data extraction to reinforce rigor of the extraction procedures.

## Conclusion

Healthcare professionals exposed to working with patients during an epidemic/pandemic are at heightened risk of mental health problems in the short and longer term. These mental health problems may interfere with the quality of patient care, although further evidence is needed. Healthcare staff need to be provided with psychosocial support to protect their mental wellbeing if they are to continue to provide high quality patient care. Few evidence-based prevention or early intervention programs exist so far. Several recommendations based on risk and protective factors of this review, as well as on additional primary studies are proposed.

## Supplementary information

**Additional file 1: Appendix 1.** Search Strategies.

## Data Availability

The selection of papers and data used to conduct this rapid review will be made available by the authors on request.

## Abbreviations

HCPs: Healthcare professionals
PTSD: Posttraumatic stress disorder
COVID-19: Coronavirus disease 2019
SARS: Severe acute respiratory syndrome
MERS: Middle East respiratory syndrome
H1N1: Influenza pandemic
H5N1: Avian influenza
CBT: Cognitive behavioral therapy
