# Supplementary material for: Psychological impact of an epidemic/pandemic on the mental health of healthcare professionals: a rapid review
Source: BMC Public Health. 2020 Aug 12;20:1230. doi: 10.1186/s12889-020-09322-z (PMC7422454; doi:10.1186/s12889-020-09322-z)
Supplement: Supplementary file 1 — Additional file 1: Appendix 1. Search Strategies. [file 12889_2020_9322_MOESM1_ESM.docx]

**Supplementary Materials**

**Appendix 1: Search Strategies**

## Embase.com

('health care personnel'/exp OR ("health care practitioner*" OR "health care professional*" OR "health care worker*" OR "health care provider*" OR "health care employee*" OR "health care volunteer*" OR "health care personnel" OR "health care staff" OR "healthcare practitioner*" OR "healthcare professional*" OR "healthcare worker*" OR "healthcare provider*" OR "healthcare staff" OR "healthcare employee*" OR "health care volunteer*" OR "healthcare personnel" OR "health worker*" OR "health personnel" OR "medical worker*" OR "hospital worker*" OR "hospital employee*" OR "hospital staff" OR "hospital personnel" OR "medical staff" OR "nursing staff" OR Physician* OR doctor OR doctors OR Nurse or nurses OR midwife OR midwives OR Humanitarian* OR "first responder*"):ti,kw) AND ('epidemic'/exp OR 'pandemic'/exp OR 'outbreak'/exp OR 'emerging infectious disease'/exp OR 'severe acute respiratory syndrome'/exp OR 'Middle East respiratory syndrome'/exp OR '2009 h1n1 influenza'/exp OR 'influenza A (H1N1)'/de OR 'influenza A (H5N1)'/de OR 'Coronavirus infection'/de OR 'SARS-related coronavirus'/exp OR 'Ebola hemorrhagic fever'/exp OR 'Zika fever'/exp OR 'West Nile fever'/de OR (Pandemic* OR "Disease outbreak*" OR "emerging infectious disease*" OR "Infectious disease epidemic*" OR 2019ncov OR "2019 ncov" OR "novel coronavirus*" OR "novel corona virus*" OR ((coronavirus* OR "corona virus*" OR "pneumonia virus*" OR cov OR ncov) AND (outbreak OR wuhan)) OR covid19 OR "covid 19" OR ((coronavirus* OR "corona virus*") AND 2019) OR sars2 OR "new coronavirus*" OR "new corona virus*" OR "ncov 2019" OR SARS OR "severe acute respiratory syndrome" OR "Middle East Respiratory Syndrome" OR MERS OR Zika OR Ebola OR "h1n1 influenza" OR "h5N1 influenza" OR "west nile fever"):ab,ti,kw) AND ('mental health'/de OR 'depression'/de OR 'depressive psychosis'/de OR 'minor depression'/de OR 'major depression'/de OR 'mixed anxiety and depression'/de OR 'reactive depression'/de OR 'anxiety'/de OR 'anxiety disorder'/de OR 'acute stress disorder'/de OR 'distress syndrome'/de OR 'posttraumatic stress disorder'/de OR 'stress'/de OR 'acute stress'/de OR 'burnout'/de OR 'professional burnout'/de OR 'adaptation syndrome'/de OR 'emotional stress'/de OR 'job stress'/exp OR 'mental stress'/de OR 'psychotrauma'/exp OR 'psychological resilience'/de OR 'coping behavior'/de OR 'psychological adjustment'/de OR 'psychological well-being'/de OR 'wellbeing'/de OR ("Mental health" OR "psychological health" OR Depression OR depressive OR Anxiet* OR anxious OR Stress OR stressed OR Trauma OR PTSD OR Coping OR "Well being" OR "Psychological impact" OR "Psychosocial impact" OR burnout OR "mental distress" OR "moral distress" OR "psychological distress" OR "adjustment disorder*" OR Emotion*):ab,ti,kw) NOT ('conference abstract'/it OR 'conference review'/it OR 'editorial'/it OR 'letter'/it) AND [2003-2020]/py AND ([english]/lim OR [french]/lim)

## PubMed

("Health Personnel"[Mesh] OR "health care practitioner*"[ti] OR "health care professional*"[ti] OR "health care worker*"[ti] OR "health care provider*"[ti] OR "health care employee*"[ti] OR "health care volunteer*"[ti] OR "health care personnel"[ti] OR "health care staff"[ti] OR "healthcare practitioner*"[ti] OR "healthcare professional*"[ti] OR "healthcare worker*"[ti] OR "healthcare provider*"[ti] OR "healthcare staff"[ti] OR "healthcare employee*"[ti] OR "healthcare volunteer*"[ti] OR "healthcare personnel"[ti] OR "health worker*"[ti] OR "health personnel"[ti] OR "medical worker*"[ti] OR "hospital worker*"[ti] OR "hospital employee*"[ti] OR "hospital staff"[ti] OR "hospital personnel"[ti] OR "medical staff"[ti] OR "nursing staff"[ti] OR Physician*[ti] OR doctor[ti] OR doctors[ti] OR Nurse[ti] OR nurses[ti] OR midwife[ti] OR midwives[ti] OR Humanitarian*[ti] OR "first responder*"[ti]) AND ("Disease Outbreaks"[Mesh] OR "Communicable Diseases, Emerging"[Mesh] OR "COVID-19" [Supplementary Concept] OR "Severe acute respiratory syndrome coronavirus 2"[Supplementary Concept] OR "Severe Acute Respiratory Syndrome"[Mesh] OR "Influenza A Virus, H1N1 Subtype"[Mesh] OR "Influenza A Virus, H5N1 Subtype"[Mesh] OR "Coronavirus Infections"[Mesh:NoExp] OR "Zika Virus Infection"[Mesh] OR "Hemorrhagic Fever, Ebola"[Mesh] OR "Middle East Respiratory Syndrome Coronavirus"[Mesh] OR "West Nile Fever"[Mesh] OR Pandemic*[tiab] OR "Disease outbreak*"[tiab] OR "emerging infectious disease*"[tiab] OR "Infectious disease epidemic*"[tiab] OR 2019ncov[tiab] OR "2019 ncov"[tiab] OR "novel coronavirus*"[tiab] OR "novel corona virus*"[tiab] OR ((coronavirus*[tiab] OR "corona virus*"[tiab] OR "pneumonia virus*"[tiab] OR cov[tiab] OR ncov) AND (outbreak[tiab] OR wuhan[tiab])) OR covid19[tiab] OR "covid 19"[tiab] OR ((coronavirus*[tiab] OR "corona virus*"[tiab]) AND 2019[tiab]) OR sars2[tiab] OR "new coronavirus*"[tiab] OR "new corona virus*"[tiab] OR "ncov 2019"[tiab] OR SARS[tiab] OR "severe acute respiratory syndrome"[tiab] OR "Middle East Respiratory Syndrome"[tiab] OR MERS[tiab] OR Zika[tiab] OR Ebola[tiab] OR "h1n1 influenza"[tiab] OR "west nile fever"[tiab]) AND ("Mental Health"[Mesh] OR "Depression"[Mesh] OR "Depressive Disorder"[Mesh:NoExp] OR "Depressive Disorder, Major"[Mesh] OR "Anxiety"[Mesh:NoExp] OR "Stress, Psychological"[Mesh] OR "Psychological Trauma"[Mesh] OR "Stress Disorders, Traumatic"[Mesh:NoExp] OR "Stress Disorders, Traumatic, Acute"[Mesh] OR "Stress Disorders, Post-Traumatic"[Mesh] OR "Resilience, Psychological"[Mesh] OR "Adaptation, Psychological"[Mesh:NoExp] OR "Emotional Adjustment"[Mesh:NoExp] OR "Psychological Distress"[Mesh] OR "Mental health"[tiab] OR "psychological health"[tiab] OR Depression[tiab] OR depressive[tiab] OR Anxiet*[tiab] OR Stress[tiab] OR Trauma[tiab] OR PTSD[tiab] OR Coping[tiab] OR "Well being"[tiab] OR "Psychological impact"[tiab] OR "Psychosocial impact"[tiab] OR burnout[tiab] OR "mental distress"[tiab] OR "moral distress"[tiab] OR "psychological distress"[tiab] OR "adjustment disorder*"[tiab] OR Emotion*[tiab]) NOT ("Editorial" [pt] OR "Letter" [pt]) AND ("2003"[dp] : "3000"[dp])

## APA PsycInfo Ovid SP

*APA PsycInfo 1806 to March Week 3 2020*

(exp health personnel/ OR ("health care practitioner*" OR "health care professional*" OR "health care worker*" OR "health care provider*" OR "health care employee*" OR "health care volunteer*" OR "health care personnel" OR "health care staff" OR "healthcare practitioner*" OR "healthcare professional*" OR "healthcare worker*" OR "healthcare provider*" OR "healthcare staff" OR "healthcare employee*" OR "health care volunteer*" OR "healthcare personnel" OR "health worker*" OR "health personnel" OR "medical worker*" OR "hospital worker*" OR "hospital employee*" OR "hospital staff" OR "hospital personnel" OR "medical staff" OR "nursing staff" OR Physician* OR doctor OR doctors OR Nurse or nurses OR midwife OR midwives OR Humanitarian* OR "first responder*").ti.) AND (exp epidemics/ OR (Pandemic* OR "Disease outbreak*" OR "emerging infectious disease*" OR "Infectious disease epidemic*" OR 2019ncov OR "2019 ncov" OR "novel coronavirus*" OR "novel corona virus*" OR ((coronavirus* OR "corona virus*" OR "pneumonia virus*" OR cov OR ncov) AND (outbreak OR wuhan)) OR covid19 OR "covid 19" OR ((coronavirus* OR "corona virus*") AND 2019) OR sars2 OR "new coronavirus*" OR "new corona virus*" OR "ncov 2019" OR SARS OR "severe acute respiratory syndrome" OR "Middle East Respiratory Syndrome" OR MERS OR Zika OR Ebola OR "h1n1 influenza" OR "h5N1 influenza" OR "west nile fever").ab,ti.) AND (exp Mental health/ OR exp major depression/ OR exp stress/ OR Anxiety/ OR anxiety sensitivity/ OR health anxiety/ OR trauma/ OR emotional trauma/ OR moral injury/ OR post-traumatic stress/ OR exp Emotions/ OR exp emotional adjustment/ OR "resilience (psychological)"/ OR exp adaptation/ OR mental disorders/ OR affective disorders/ OR anxiety disorders/ OR chronic mental illness/ OR dissociative disorders/ OR eating disorders/ OR psychosis/ OR sleep wake disorders/ OR "stress and trauma related disorders"/ OR thought disturbances/ OR ("Mental health" OR "psychological health" OR Depression OR depressive OR Anxiet* OR anxious OR Stress OR stressed OR Trauma OR PTSD OR Coping OR "Well being" OR "Psychological impact" OR "Psychosocial impact" OR burnout OR "mental distress" OR "moral distress" OR "psychological distress" OR "adjustment disorder*" OR Emotion*).ab,ti.)

## Web of Science – Core collection^[[1]](#footnote-1)^

TS=(("health care practitioner*" OR "health care professional*" OR "health care worker*" OR "health care provider*" OR "health care employee*" OR "health care personnel" OR "health care staff" OR "health care employee*" OR "health care volunteer*" OR "healthcare practitioner*" OR "healthcare professional*" OR "healthcare worker*" OR "healthcare provider*" OR "healthcare staff" OR "healthcare employee*" OR "healthcare volunteer*" OR "healthcare personnel" OR "health worker*" OR "health personnel" OR "medical worker*" OR "hospital worker*" OR "hospital employee*" OR "hospital staff" OR "hospital personnel" OR "medical staff" OR "nursing staff" OR Physician* OR "doctor" OR "doctors" OR "Nurse" OR "nurses" OR "midwife" OR "midwives" OR "Humanitarian*" OR "first responder*") AND ("Pandemic*" OR "Disease outbreak*" OR "emerging infectious disease*" OR "Infectious disease epidemic*" OR "2019ncov" OR "2019 ncov" OR "novel coronavirus*" OR "novel corona virus*" OR (("coronavirus*" OR "corona virus*" OR "pneumonia virus*" OR "cov" OR "ncov") AND ("outbreak" OR "wuhan")) OR "covid19" OR "covid 19" OR (("coronavirus*" OR "corona virus*") AND "2019") OR "sars2" OR "new coronavirus*" OR "new corona virus*" OR "ncov 2019" OR "SARS" OR "severe acute respiratory syndrome" OR "Middle East Respiratory Syndrome" OR "MERS" OR "Zika" OR "Ebola" OR "h1n1 influenza" OR "h5N1 influenza" OR "west nile fever") AND ("Mental health" OR "psychological health" OR "psychological impact" OR "psychosocial impact" OR "Depression" OR "depressive" OR Anxiet* OR "anxious" OR "Stress" OR "stressed" OR "Trauma" OR "PTSD" OR "Coping" OR "Well being" OR "burnout" OR "mental distress" OR "moral distress" OR "psychological distress" OR "adjustment disorder*" OR "Emotion*"))

## Results of the search strategies

| **Database** | **Date of the search** | **Number of found articles** |
| --- | --- | --- |
| PubMed | March 22, 2020 | 241 |
| Embase.com | March 22, 2020 | 578 |
| *APA PsycInfo Ovid SP 1806 to March Week 3 2020* | March 22, 2020 | 129 |
| Web of Science – Core collection | March 22, 2020 | 333 |
| Total |  | 1281 |

1. Core Collection includes: Science Citation Index Expanded (1900-present), Social Sciences Citation Index (1900-present), Arts & Humanities Citation Index (1975-present), Conference Proceedings Citation Index-Science (1990-present), Conference Proceedings Citation Index-Social Science & Humanities (1990-present), Book Citation Index– Science (2005-present), Book Citation Index– Social Sciences & Humanities (2005-present), Emerging Sources Citation Index (2015-present), Current Chemical Reactions (1985-present), (Includes Institut National de la Propriete Industrielle structure data back to 1840), Index Chemicus (1993-present) [↑](#footnote-ref-1)
